# 伴弥漫大B细胞成分的初诊滤泡淋巴瘤患者的临床特征及生存

**DOI:** 10.3760/cma.j.issn.0253-2727.2022.06.003

**Published:** 2022-06

**Authors:** 志娟 林, 洁 查, 树华 易, 志峰 李, 凌燕 平, 晓华 何, 海峰 余, 重 郑, 卫 徐, 菲莉 陈, 颖 谢, 碧云 陈, 会来 张, 莉 王, 凯阳 丁, 文瑜 李, 海燕 杨, 维莅 赵, 录贵 邱, 志铭 李, 玉琴 宋, 兵 徐

**Affiliations:** 1 厦门大学第一附属医院血液科，厦门大学医学院血液研究所，厦门市恶性血液病诊断与治疗重点实验室，厦门 361003 Department of Hematology, the First Affiliated Hospital of Xiamen University and Institute of Hematology, School of Medicine, Xiamen University, Key Laboratory of Xiamen for Diagnosis and Treatment of Hematological Malignancy, Xiamen 361003, China; 2 中国医学科学院血液病医院（中国医学科学院血液学研究所），实验血液学国家重点实验室，国家血液系统疾病临床医学研究中心，天津 300020 State Key Laboratory of Experimental Hematology, National Clinical Research Center for Blood Diseases, Institute of Hematology & Blood Diseases Hospital, Chinese Academy of Medical Sciences & Peking Union Medical College, Tianjin 300020, China; 3 北京大学肿瘤医院，恶性肿瘤发病机制与转化研究教育部重点实验室，北京 100142 Key Laboratory of Carcinogenesis and Translational Research (Ministry of Education), Peking University Cancer Hospital & Institute, Beijing 100142, China; 4 中山大学肿瘤防治中心，广州 510060 Department of Medical Oncology, State Key Laboratory of Oncology in South China, Collaborative Innovation Center for Cancer Medicine, Sun Yat-sen University Cancer Center, Guangzhou 510060, China; 5 中国科学院大学附属肿瘤医院（浙江省肿瘤医院）淋巴瘤科，杭州 310022 Department of Lymphoma, Cancer Hospital of the University of Chinese Academy of Sciences (Zhejiang Cancer Hospital), Hangzhou 310022, China; 6 上海血液学研究所，医学基因组学国家重点实验室，国家转化医学研究中心，上海交通大学医学院附属瑞金医院，上海 200025 Shanghai Institute of Hematology, State Key Laboratory of Medical Genomics, National Research Center for Translational Medicine at Shanghai, Ruijin Hospital Affiliated to Shanghai Jiao Tong University School of Medicine, Shanghai 200025, China; 7 南京医科大学第一附属医院，江苏省人民医院血液科，南京 210029 Department of Hematology, The First Affiliated Hospital of Nanjing Medical University, Jiangsu Province Hospital, Nanjing 210029, China; 8 广东省医学科学院广东省人民医院淋巴瘤科，广州 510080 Lymphoma Division, Guangdong Provincial People's Hospital, Guangdong Academy of Medical Sciences, Guangzhou 510080, China; 9 福建医科大学福建省立医院血液科，福州 350001 Shengli Clinical Medical College of Fujian Medical University, Department of Hematology, Fujian Provincial Hospital, Fujian Medical University, Fuzhou 350001, China; 10 天津医科大学肿瘤医院淋巴瘤科，天津 300060 Department of Lymphoma, Tianjin Medical University Cancer Hospital, Tianjin 300060, China; 11 中国科学技术大学附属第一医院（安徽省立医院）血液科，合肥 230001 Department of Hematology, The First Affiliated Hospital of USTC Anhui Provincial Hospital, Hefei 230001, China

**Keywords:** 淋巴瘤，滤泡性, 淋巴瘤，大B细胞，弥漫性, 中国, Lymphoma, follicular, Lymphoma, large B-cell, diffuse, China

## Abstract

**目的:**

探索初诊伴弥漫大B细胞成分的滤泡淋巴瘤（FL）患者的临床特征及生存。

**方法:**

纳入国内11个医学中心2000−2020年间分级1～3a级、年龄≥18岁初诊FL患者1 845例，筛选伴弥漫大B细胞成分的患者。回顾性分析患者的临床资料及生存数据，应用单因素及多因素分析筛选影响预后的因素。

**结果:**

146例（7.9％）初诊FL患者病理伴弥漫大B细胞成分，中位年龄56（25～83）岁，男79例（54.1％）。127例患者病理提示弥漫大B细胞成分比例，依据弥漫大B细胞比例是否≥50％将患者分为2组。研究发现，弥漫大B细胞成分≥50％患者较弥漫大B细胞成分<50％患者有更高的3级比例（94.3％对91.9％，*P*＝0.010）、Ki-67指数≥70％比例（58.5％对32.9％，*P*＝0.013）及PET-CT的SUVmax≥13比例（72.4％对46.3％，*P*＝0.030）。所有患者均接受CHOP或CHOP样±利妥昔单抗方案化疗，总反应率（ORR）为88.2％，完全缓解（CR）率为76.4％。在不同比例弥漫大B细胞成分组中，诱导治疗后缓解率及治疗后2年内疾病进展（POD24）发生率的差异均无统计学意义（*P*值均>0.05）。总体预计5年无进展生存（PFS）率为58.9％，5年总生存（OS）率为90.4％，POD24患者的5年OS率较非POD24患者下降（70.3％对98.5％，*P*<0.001）。与利妥昔单抗不维持治疗相比，利妥昔单抗维持治疗不能使患者的5年PFS率获益（57.7％对58.8％，*P*＝0.543），5年OS率具有获益趋势，但差异无统计学意义（100％对87.8％，*P*＝0.082）。多因素分析显示，诱导治疗后未达到CR是影响患者PFS的独立危险因素（*P*＝0.006），而LDH高于正常值是影响患者OS的独立危险因素（*P*＝0.031）。

**结论:**

伴弥漫大B细胞成分≥50％的FL患者，临床及病理特征更具侵袭性，CHOP/CHOP样±利妥昔单抗方案可以改善患者的临床疗效，利妥昔单抗维持治疗不能使患者的PFS和OS获益，诱导治疗后未达到CR是影响患者PFS的独立危险因素。

## Abstract (en)

**Objective:**

To explore the clinical features and survival of newly diagnosed follicular lymphoma (FL) patients with diffuse large B-cell lymphoma (DLBCL) component.

**Methods:**

1845 newly diagnosed FL patients aged ≥ 18 years with grades 1−3a in 11 medical centers in China from 2000 to 2020 were included, and patients with DLBCL component were screened. The clinical data and survival data of the patients were retrospectively analyzed, and the prognostic factors were screened by univariate and multivariate analysis.

**Results:**

146 patients (7.9％) with newly diagnosed FL had DLBCL component. The median age was 56 (25−83) years, 79 males (54.1％). The pathology of 127 patients showed the proportion of DLBCL component. Patients were divided into two groups according to whether the proportion of DLBCL component was ≥ 50％. The study found that patients with DLBCL component ≥ 50％ had higher grade 3 ratio (94.3％ *vs* 91.9％, *P*＝0.010), Ki-67 index ≥ 70％ ratio (58.5％ *vs* 32.9％, *P*＝0.013) and PET-CT SUVmax ≥ 13 ratio (72.4％ *vs* 46.3％, *P*＝0.030) than patients with DLBCL component<50％. All patients received CHOP or CHOP like ± rituximab chemotherapy. The overall response rate (ORR) was 88.2％, and the complete response (CR) rate was 76.4％. In the groups with different proportions of DLBCL component, there was no significant difference in the remission rate after induction treatment and the incidence of disease progression within 2 years after initiation of treatment (POD24) (*P*<0.05). The overall estimated 5-year progression free survival (PFS) rate was 58.9％, and the 5-year overall survival (OS) rate was 90.4％. The 5-year OS rate of POD24 patients was lower than that of non POD24 patients (70.3％ *vs* 98.5％, *P*<0.001). Compared with non maintenance treatment of rituximab, maintenance treatment of rituximab could not benefit the 5-year PFS rate (57.7％ *vs* 58.8％, *P*＝0.543), and the 5-year OS rate had a benefit trend, but the difference was not statistically significant (100％ *vs* 87.8％, *P*＝0.082). Multivariate analysis showed that failure to reach CR after induction treatment was an independent risk factor for PFS (*P*＝0.006), while LDH higher than normal was an independent risk factor for OS (*P*＝0.031).

**Conclusion:**

FL patients with DLBCL component ≥50％ have more invasive clinical and pathological features. CHOP/CHOP like ± rituximab regimen can improve the clinical efficacy of patients. Rituximab maintenance therapy can not benefit the PFS and OS of patients. Failure to reach CR after induction therapy was the independent unfavorable factor for PFS.

滤泡淋巴瘤（follicular lymphoma，FL）起源于生发中心B细胞，其发病率在中国B细胞淋巴瘤中约占8％，是最常见的惰性B细胞淋巴瘤[1]。本中心前期针对中国FL患者的回顾性分析显示，中国FL患者预后良好，5年总生存（OS）率为89％，5年无进展生存（PFS）率为61％[2]，与欧美国家类似[3]。但每年有约3％ FL患者在病程中转化为更具侵袭性的淋巴瘤，持续至少15年，此后转化风险逐渐下降。FL转化为弥漫大B细胞淋巴瘤（diffuse large B cell lymphoma，DLBCL）最常见，主要的分子机制为“线性”或“非线性”遗传学克隆演变[4]。既往多项研究显示，治疗后出现转化的患者预后不良[1]–[3],[5]，5年OS率仅65％[2]。

小于10％ FL患者在初诊时即可合并DLBCL成分[6]–[8]。有研究报道，在878例初诊FL患者中，约40例（4.56％）患者伴DLBCL成分，其中86％为生发中心来源，10％为活化细胞来源，4％为不能分型。同时，这部分患者中有7％为“双打击”，即荧光原位杂交（FISH）检出MYC伴Bcl-2或Bcl-6易位。这部分患者的PFS及OS均介于FL和DLBCL之间[7]。然而，中国尚无针对此类人群的研究。因此，本研究在中国FL工作组的帮助下进行一项真实世界研究，旨在探索初诊伴弥漫大B细胞成分的FL患者的临床特征及生存。

## 病例与方法

1. 病例：本研究的病例来自国内11个医学中心，分别是北京大学附属肿瘤医院、福建省省立医院、广东省人民医院、江苏省人民医院、上海交通大学医学院附属瑞金医院、天津医科大学肿瘤医院、厦门大学附属第一医院、中国科学技术大学附属第一医院（安徽省立医院）、中国医学科学院血液病医院、浙江省肿瘤医院、中山大学肿瘤防治中心。纳入11个研究中心2000−2020年间1～3a级、年龄≥18岁的初诊FL患者1 845例，所有患者均经病理组织检查和免疫组织化学染色确诊，从中筛选合并DLBCL成分的患者146例。

2. 临床资料：收集患者的一般情况（年龄、性别以及体能状态等）、临床特征（病理分级、分期、DLBCL成分比例、治疗方案、疗效）及生存数据等。患者的Ki-67分层、DLBCL成分比例分层及PET-CT的SUVmax值分层参照文献[7]–[10]。虽然目前世界卫生组织（WHO）病理分组根据滤泡成分和弥漫成分所占比例将FL分为：①滤泡为主型（滤泡比例>75％）；②滤泡和弥漫混合型（滤泡比例25％～75％）；③局灶滤泡型（滤泡比例<25％）。但我们参照文献[7]–[8]的方法，以50％为界将患者分为两组以便于研究统计。

3. 疗效：治疗疗效参考Lugano 2007标准进行评估[11]。生存数据主要关注有无治疗后2年内疾病进展（POD24）及PFS、OS[12]。PFS定义为开始治疗至发生疾病进展或任何原因死亡的时间。OS定义为开始治疗至因任何原因死亡的时间。

4. 随访：采用电话、门诊复查和住院复查的方式进行随访，随访截止日期为2020年12月31日，中位随访时间30.7（0.3～236.8）个月。

5. 统计学处理：本研究采用SPSS 24.0软件进行统计学分析。计数资料用例数（百分比）表示，组间比较采用列联表*χ*^2^检验。生存分析采用Kaplan-Meier法，并绘制Kaplan-Meier生存曲线。单因素生存分析采用Log-rank检验。将单因素分析中*P*<0.10的因素纳入多因素Cox模型，并确定各个因素的*HR*、95％ *CI*、*P*值。双侧*P*<0.05为差异有统计学意义。

## 结果

1. 临床特征：1 845例初诊FL患者中，146例（7.9％）病理伴有DLBCL成分，患者中位年龄56（25～83）岁，患者的基本临床资料见表1。

146例患者中，16例病理未提示具体DLBCL成分比例，另有3例通过不同解剖部位病理分别诊断为FL和DLBCL，其余127例患者提示了具体DLBCL成分比例，以DLBCL成分比例是否≥50％将患者分为2组（表1）。其中41.7％的患者伴≥50％的DLBCL成分。两组患者的性别、年龄差异无统计学意义。与伴<50％DLBCL成分的患者相比，伴≥50％ DLBCL成分患者3级比例更高（*P*＝0.010），具有更高的Ki-67指数（*P*＝0.013）及PET-CT的SUVmax（*P*＝0.030）（表1）。

**表1:** 伴弥漫大B细胞淋巴瘤（DLBCL）成分滤泡淋巴瘤（FL）患者的临床特征［例（％）］

特征	总体（146例）	DLBCL成分<50%（74例）	DLBCL成分≥50%（53例）	*χ*^2^值	*P*值
男性	79（54.1）	38（52.8）	33（62.3）	2.093	0.351
年龄				0.069	0.966
<60岁	94（64.4）	46（62.2）	34（64.2）		
≥60岁	52（35.6）	28（37.8）	19（35.8）		
分级				16.753	0.010
1～2	12（8.2）	6（8.1）	3（5.7）		
3a	57（39.0）	29（39.2）	21（39.6）		
3b	54（37.0）	31（41.9）	18（34.0）		
未知	23（15.8）	8（10.8）	11（20.8）		
Ki-67指数^a^				8.641	0.013
<70%	81（57.0）	47（67.1）	22（41.5）		
≥70%	61（43.0）	23（32.9）	31（58.5）		
B症状^a^				1.074	0.584
无	66（74.2）	39（73.6）	19（73.1）		
有	23（25.8）	14（26.4）	7（26.9）		
ECOG评分^a^				18.051	0.006
<2分	124（91.9）	56（83.6）	50（100）		
≥2分	11（8.1）	11（16.4）	0（0）		
Ann Arbor分期				1.017	0.715
Ⅰ～Ⅱ	52（38.3）	26（37.6）	19（38.8）		
Ⅲ～Ⅳ	84（61.7）	43（62.4）	30（61.2）		
LDH>正常值^a^				0.765	0.682
无	71（51.1）	35（50.7）	23（45.1）		
有	68（48.9）	34（49.3）	28（54.9）		
骨髓累及^a^				2.520	0.284
无	122（84.7）	60（81.1）	46（90.2）		
有	22（15.3）	14（18.9）	5（9.8）		
PET-CT^a^				4.715	0.030
SUVmax<13	35（43.8）	22（53.7）	8（27.6）		
SUVmax≥13	45（56.3）	19（46.3）	21（72.4）		
FL国际预后指数				1.483	0.830
低危	66（45.2）	32（43.2）	25（47.2）		
中危	38（26.0）	19（25.7）	13（24.5）		
高危	42（28.8）	23（31.1）	15（28.3）		

注：^a^部分患者信息缺失；ECOG：美国东部肿瘤协作组

2. 治疗及疗效：在有详细治疗信息的142例患者中（表2），所有患者均接受CHOP/CHOP样±R方案化疗，12.0％患者在诱导及维持治疗阶段均未接受利妥昔单抗治疗，66.2％患者仅在诱导治疗阶段接受利妥昔单抗治疗。21.8％患者在诱导治疗结束后接受维持治疗。在进行疗效评估的127例患者中，诱导治疗的总反应率（ORR）为88.2％，其中完全缓解（CR）率76.4％，部分缓解（PR）率11.8％。142例患者中有38例（26.8％）患者出现POD24。在不同比例DLBCL成分组中，诱导治疗疗效及POD24发生率差异无统计学意义（表2）。

**表2:** 伴弥漫大B细胞淋巴瘤（DLBCL）成分滤泡淋巴瘤（FL）患者的治疗及疗效［例（％）］

治疗及疗效	总体（142例）	DLBCL成分<50%（73例）	DLBCL成分≥50%（51例）	*χ*^2^值	*P*值
维持治疗				0.627	0.731
无	111（78.2）	55（75.3）	41（80.4）		
有	31（21.8）	18（24.7）	10（19.6）		
利妥昔单抗				4.369	0.627
无	17（12.0）	13（17.8）	4（7.8）		
诱导治疗	94（66.2）	42（57.5）	37（72.5）		
维持治疗	3（2.1）	2（2.7）	1（2.0）		
诱导治疗+维持治疗	28（19.7）	16（21.9）	9（17.6）		
诱导治疗疗效^a^				4.085	0.665
CR	97（76.4）	50（76.9）	32（71.1）		
PR	15（11.8）	9（13.8）	4（8.9）		
SD	6（4.7）	3（4.6）	3（6.7）		
PD	9（7.1）	3（4.6）	6（13.3）		
含利妥昔单抗诱导方案疗效			2.413	0.878
CR	70（72.9）	41（74.5）	29（70.7）		
PR	12（12.5）	8（14.5）	4（9.8）		
SD+PD	14（15.6）	6（11.0）	8（19.5）		
POD24				0.652	0.722
无	104（73.2）	54（75.0）	35（68.6）		
有	38（26.8）	18（25.0）	16（31.4）		

注：^a^部分患者信息缺失；CR：完全缓解；PR：部分缓解；SD：疾病稳定；PD：疾病进展；POD24：治疗后2年内疾病进展

3. 预后：预计总体5年PFS率为58.9％，5年OS率为90.4％，出现POD24患者的5年OS率较无POD24患者下降（70.3％对98.5％，*P*<0.001）。与利妥昔单抗不维持治疗相比，利妥昔单抗维持治疗不能使患者的5年PFS率获益（57.7％对58.8％，*P*＝0.539），OS率具有获益趋势，但差异无统计学意义（100％对87.8％，*P*＝0.082）。在不同DLBCL成分组中，有无维持治疗组的PFS及OS差异均无统计学意义（*P*值均>0.05）。DLBCL成分<50％患者中，不维持治疗组与维持治疗组的5年OS率分别为84.7％和100％（*P*＝0.130），5年PFS率分别为65.2％和70.2％（*P*＝0.663）。DLBCL成分≥50％患者中，不维持治疗组与维持治疗组的5年OS率分别为87.7％和100％（*P*＝0.310），5年PFS率分别为57.9％和64.0％（*P*＝0.371）。

单因素分析结果显示患者Ann Arbor分期Ⅲ～Ⅳ期、LDH>正常值、骨髓累及、初诊时PET-CT SUVmax≥13，FL国际预后指数（FLIPI）高危及诱导治疗后未达到CR是影响患者PFS的不良预后因素（*P*值均<0.10）。而LDH>正常值、骨髓累及、FLIPI高危及未接受维持治疗是影响患者OS的不良预后因素（*P*值均<0.10）（表3）。将上述因素纳入多因素分析，结果显示，诱导治疗后未达到CR是影响患者PFS的独立危险因素（*HR*＝3.60，95％*CI* 1.46～8.85，*P*＝0.006），而LDH>正常值是影响患者OS的独立危险因素（*HR*＝11.70，95％*CI* 1.26～109.32，*P*＝0.031）。Ann Arbor分期Ⅲ～Ⅳ期（*P*＝0.049）、LDH>正常值（*P*＝0.001）、初诊时PET-CT SUVmax≥13（*P*＝0.014）、FLIPI高危（*P*＝0.001）及诱导治疗后未达到CR（*P*<0.001）的患者更易出现POD24。

**表3:** 影响伴DLBCL成分FL患者无进展生存（PFS）和总生存（OS）的单因素分析

因素	PFS			OS		
	5 年PFS率（%）	*χ*^2^值	*P*值	5 年OS率（%）	*χ*^2^值	*P*值
性别		0.330	0.566		0.014	0.906
男	62.4			91.8		
女	54.2			89.0		
年龄		<0.001	0.995		1.824	0.177
<60岁	64.4			93.8		
≥60岁	64.9			84.6		
分级		4.025	0.259		3.625	0.305
1～2	30.9			80.2		
3a	55.6			88.1		
3b	69.6			92.1		
未知	60.7			100.0		
Ki-67指数		0.113	0.736		0.009	0.926
<70%	60.1			91.0		
≥70%	55.8			90.9		
DLBCL成分比例		1.528	0.466		0.506	0.776
<50%	66.8			88.5		
≥50%	58.0			90.1		
Ann Arbor分期		4.531	0.033		1.342	0.247
Ⅰ～Ⅱ	72.2			93.4		
Ⅲ～Ⅳ	50.2			88.6		
LDH>正常值		14.977	<0.001		8.892	0.003
无	74.5			97.1		
有	42.3			81.9		
骨髓累及		6.085	0.014		4.863	0.027
无	63.1			92.4		
有	39.2			75.6		
PET-CT		3.732	0.053		2.423	0.120
SUVmax<13	77.5			100.0		
SUVmax≥13	39.6			88.6		
FLIPI		12.593	0.002		5.045	0.080
低危	75.3			94.6		
中危	49.7			91.7		
高危	41.8			81.8		
诱导治疗达CR		38.706	<0.001		2.176	0.140
无	10.3			84.4		
有	73.6			91.6		
维持治疗		0.377	0.539		3.024	0.082
无	58.8			87.8		
有	57.7			100.0		
利妥昔单抗		1.028	0.795		3.100	0.376
无	67.3			86.2		
诱导阶段	56.6			89.8		
维持治疗	50.0			100.0		
诱导治疗+维持治疗	57.6			100.0		

注：DLBCL：弥漫大B细胞淋巴瘤；FL：滤泡淋巴瘤；FLIPI：FL国际预后指数；CR：完全缓解

## 讨论

本研究显示，7.9％初诊FL患者病理伴DLBCL成分，与国外报道相似[7]–[8]。本研究患者的中位年龄56（25～83）岁，35.6％患者年龄≥60岁，年龄低于国外类似报道。男性患者比例54.1％，低于国外报道（60％～70％）。本研究1～2级患者仅8.2％，低于既往新加坡研究报道的26.4％[8]。Ann Arbor分期Ⅲ～Ⅳ期患者比例与国外报道相似，但本研究患者出现骨髓累及的比例（15.3％）显著低于国外报道（27％～33％）。这一差异可能由于本研究患者诊断时间跨度大，既往判断骨髓累及的方式大部分通过骨髓活检手段，近年来才逐渐通过联合骨髓流式细胞术和PCR等更敏感的方案判断有无骨髓累及，导致部分患者可能漏诊。

为了探讨不同DLBCL成分比例患者的基线特征、病理特征及预后是否存在差异，我们参照既往报道[7]–[8]的方法，根据DLBCL成分比例是否≥50％将患者分为两组进行比较。结果显示两组患者性别、年龄、Ann Arbor分期等信息的差异均无统计学意义。本研究发现合并≥50％ DLBCL成分患者3级比例更高（*P*＝0.01），在初诊时具有更高的Ki-67指数（*P*＝0.013）及PET-CT的SUVmax（*P*＝0.030）。提示合并更高比例DLBCL成分的患者在临床以及病理特征上更具侵袭性。

研究显示，不同DLBCL成分比例、Ki-67指数患者的治疗有效率（ORR及CR率）及生存率（PFS率及OS率）差异均无统计学意义，与既往文献报道相似。Lim等[8]的研究显示，Bcl-6±MYC重排会显著影响患者的OS，PFS的差异无统计学意义。患者的DLBCL成分比例、Ki-67指数、细胞起源、Bcl-2、Bcl-6及MYC表达量均不影响患者的生存。

在诱导治疗方面，根据NCCN指南及中国FL专家共识，FL的初诊治疗方案为R-CHOP/R-CVP方案或BR方案（苯达莫司汀联合抗CD20单抗）。由于苯达莫司汀直至2018年12月才在中国上市，因此，本研究有治疗信息的142例患者均采用CHOP/CHOP样±R方案化疗。有疗效评估的127例患者中，CHOP±R或CHOP样±R方案化疗可以使患者的ORR达到88.2％，CR率达到76.4％。西班牙一项研究纳入40例患者，98％接受R-CHOP/CVP方案，ORR以及CR率分别为80％和65％[7]。另外，在探讨FL初诊治疗方案的GALLIUM研究中，R-CHOP方案达到的ORR及CR率分别为88％和61％[13]。这些数据提示，本研究人群达到的ORR及CR率与单纯FL患者相似，优于既往类似报道。

既往研究提示FL患者接受利妥昔单抗维持治疗可以使PFS时间显著延长[14]，而DLBCL患者并不能从维持治疗中获益[15]。加拿大的一项回顾性研究显示，利妥昔单抗维持治疗同样不能使合并DLBCL成分的FL患者的生存获益[16]。但这项研究并未探讨不同DLBCL成分比例对患者维持治疗的影响。本研究显示，无论DLBCL成分比例如何，维持治疗均不能使患者的PFS及OS获益。但本研究及上述加拿大研究均为回顾性研究，需要严格的随机对照研究进一步验证。

本研究显示此类患者的5年OS率达到90.4％，优于既往类似报道（71.2％～73％）。而5年PFS率达58.9％，与既往报道相似（55％～61.6％）。既往尚无研究探讨POD24对此类患者预后的影响。本研究显示，与单纯FL类似，在合并DLBCL成分的FL患者中，POD24同样提示预后不良。既往新加坡一项研究发现B症状、诱导治疗后未达到CR及化疗方案是影响患者生存的不良预后因素[8]，但本研究发现诱导治疗后未达到CR是影响患者PFS的独立不良因素，而LDH高于正常值是影响患者OS的独立不良因素。出现上述差异的原因可能是：本研究伴B症状患者比例低于既往报道（25.3％对30.2％）；本研究所有患者均接受CHOP/CHOP样±R方案化疗。

综上，本研究首次探讨了初诊伴DLBCL成分的中国FL患者的临床特征和生存。结果提示，伴DLBCL成分比例≥50％的患者在临床及病理特征上更具侵袭性。CHOP/CHOP样±R方案可提高此类患者的临床疗效，而利妥昔单抗的维持治疗不能使患者的PFS和OS获益。诱导治疗后未达到CR是影响此类患者PFS的独立危险因素。
